# Primary Urachal Actinomycosis: Case Report and Literature Review

**DOI:** 10.5812/numonthly.10933

**Published:** 2013-11-13

**Authors:** Ghodratolah Maddah, Behzad Feizzdeh Kerigh, Nema Mohamadian, Vafa Bagheri

**Affiliations:** 1 Endoscopic and Minimally Invasive Surgery Research Center, Mashhad University of Medical Sciences, Mashhad, IR Iran; 2 Department of Urology, Mashhad University of Medical Sciences, Mashhad, IR Iran; 3 Deprtment of Pathology, Mashhad University of Medical Sciences, Mashhad, IR Iran; 4 Deprtment of Surgery, Mazandaran University of Medical Sciences, Sari, IR Iran

**Keywords:** Urachal Cyst, Actinomycosis, Abdominal Pain

## Abstract

Actinomycosis can involve all parts of the urogenital system. Urachal actinomycosis rarely reported and was mistaken with urachal adenocarcinoma. We report a case of urachal actinomycosis that presented with abdominal pain and underwent laparotomy with the diagnosis of urachal malignancy pathology reviewed the diagnosis of urachal actinomycosis. Patient had no problem in two years follow up.

## 1. Introduction

Actinomycosis is an infectious, chronic and granulomatous disease caused by gram-positive non-aerobic bacteria. These bacteria are te natural flora of mouth cavity especially teeth and tonsil, GI especially appendix and colon, and genital system in human that rarely causes disease. The prevalence of clinical actinomycosis is cervicofacial 50%, abdominal 20%, and thoracic 15%, respectively (1, 2).

Primary urachus actinomycosis is rarely reported in English literature and is usually mistaken with urachus malignancy (3, 4). Here, we report a primary urachus actinomycosis in a young man.

## 2. Case Report

A 23-years old man, the worker of granite mine referred complaining of intermittent pain around the umbilicus from several months ago. The pain was progressive and continuous from 6 months ago. Recently, the patient had umbilicus pain during voiding. The patient didn’t mention the symptoms of weight and appetite loss During examination, a solid and a little tender mass was tangible as about 12 cm expanding from umbilicus to hypogastria. Fothergil test (the mass is better tangible when the feet were above) was positive. He had no fever and the examination of other parts of his body was normal.

**Figure 1.**
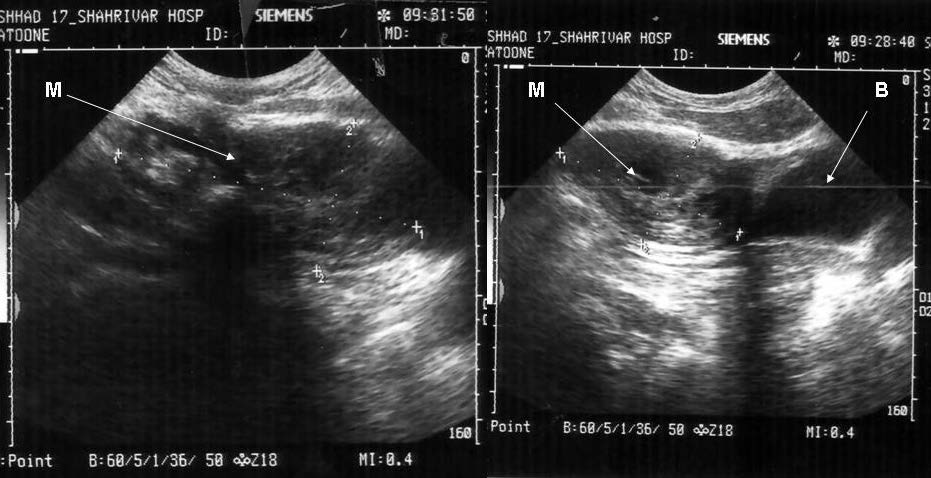
Heterogeneous Mass in the Sonographic Examination M: Mass, B: Bladder

**Figure 2.**
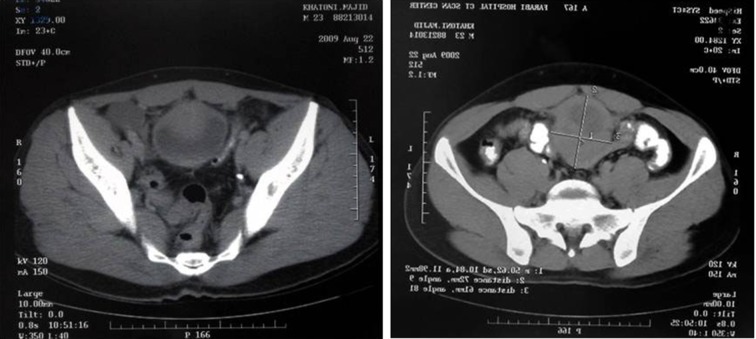
CT Scanning Showed Solid Mass Above the Bladder

Sonography showed the heterogeneous mass with size of 124 × 61 mm expanding from the umbilicus to the bladder (Figure 1). Abdominal and pelvic CT scan showed a solid mass beginning from the umbilicus with pressure on the bladder (Figure 2). Chest X-Ray was normal. Laboratory tests of urine, urea, creatinine, and liver function tests were normal. He didn’t have leukocytosis and anemia. 

The patient underwent cystoscopy due to suspected urachus adenocarcinoma. Urothelium was intact but mass pressure on bladder was observed. The patient underwent laparotomy with the incision of under umbilicus middle line. The mass had adherence to bladder and omentum and posterior rectus sheath which was removed with suitable margin along with partial cystectomy. The mass was solid, irregular, and brown. Bilateral lymphadenectomy was performed due to gross lymph nodes on iliac vessels.

**Figure 3.**
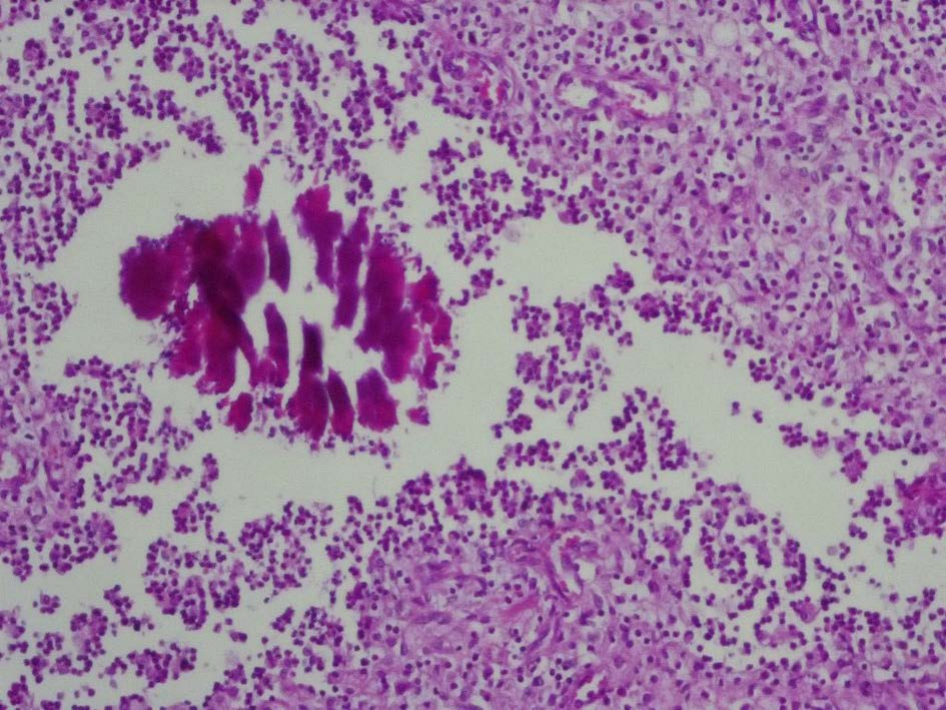
Sulphur Granules of Actinomyces With Peripheral Mixed Inflammatory Reaction. H&E Staining, 100X

**Figure 4.**
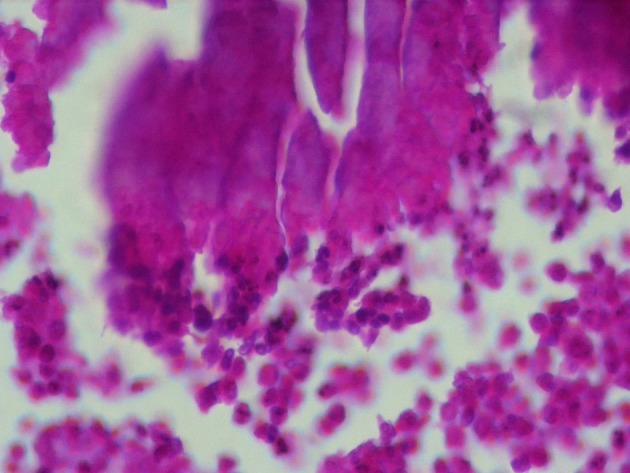
Sulphur Granules of Actinomyces With Peripheral Mixed Inflammatory Reaction. H&E Staining, 400X

**Figure 5.**
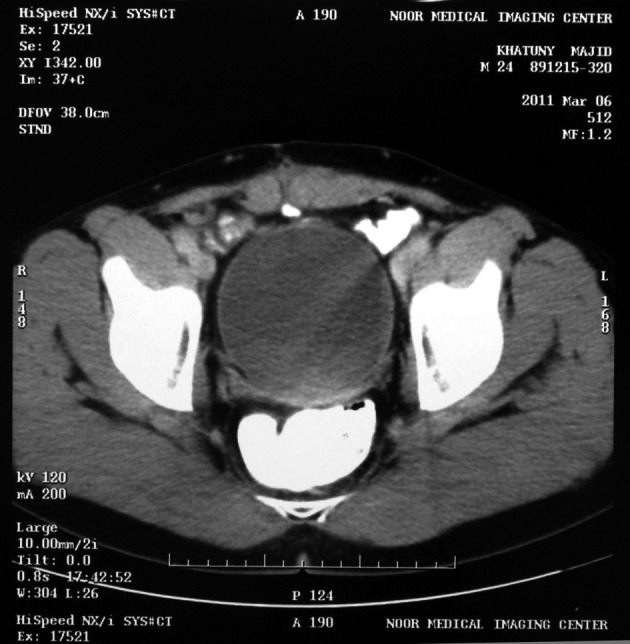
Normal CT Scanning After Two Years Follow-up.

Microscopic evaluation of mass reported the actinomycosis without involvement of bladder urothelium (Figures 3 and 4). The lymph nodes were reactive. After diagnosis, Amoxicillin was administered for 8 weeks. The patient didn’t have any problem at 2-years follow-up (Figure 5). 

## 3. Discussion

Actinomycosis is a chronic, indolent and recurrent disease which can involve all urogenital sections such as kidney, bladder, prostate, and testicle (5). Abdominal and pelvic actinomycosis is usually caused in women due to use of intrauterine device, but other risk factors such as abdominal surgery, viscus rupture, tubo-ovarian abscess, diabetes, use of steroid and neoplasm are also were reported (6-8). It was emphasis that actinomycosis can complicate urachus through umbilicus and its abnormalities such as cyst and sinus that susceptible it to non-aerobic infection (9). Our patient didn’t have any of these risk factors.

Urachus actinomycosis is rarely reported and the patients usually refer with symptoms of tangible mass under umbilicus, abdominal pain, weight loss, appetite decrease, fever, and laboratory signs such as leukocytosis and anemia (10). Our patient also referred with abdominal and voiding pain, but didn’t have laboratory signs such as leukocytosis and anemia.

Correct diagnosis before surgery is reported in only lower than 10% of cases. Abdominal actinomycosis may be mistaken with malignancy, bowel tuberculosis, crohn disease, diverticular and rectus sheath pathologies. Malignancy and abscess are in differential diagnosis (11). In our patient, mass was removed with possible diagnosis of tumor and partial cystectomy and bilateral lymphadenectomy were performed and diagnosis was determined after surgery.

Imaging findings are non-specific in urachal actimycosis. Sonography determines the solid or cystic mass but may underestimate the expansion of inflammatory reaction. According to most reports, CT scan can better determine the status of mass than MRI (magnetic resonance imaging). The findings of PET/CT of actinomycosis are as intense hypermetabolism which is similar to malignancy (3, 12). Low signal intensity in MRI at T2-weighted sequences may suggest the actinomycosis (4). In our patient, sonography and CT-scan didn’t help to definite diagnosis and only one mass suspicious to malignancy was suggested.

In a study about pelvic actinomycosis in 33 cases, cystoscopy was performed on 12 patients and the findings of cystoscopy reported the pressure effect of external mass on bladder, bullosis edema, and vegetative proliferation in one case showing chronic inflammatory changes (8). Furthurmore, in our patient, cystoscopy was performed due to voiding pain and the possibility of urach adenocarcinoma; urothelium was intact but pressure of mass on bladder was observed.

Since the clinical laboratory and radiologic signs are not special, in most cases, the diagnosis is determined after surgery (9). If the diagnosis is performed before surgery, Penicillin is the drug of choice which should be intravenously administered and then oral Penicillin and Amoxicillin should be administered for a long time (13). However, some researchers believe that combination therapy of both surgery and antibiotic administration is the most effective modality and good results are observed in more than 90% of cases (5, 14). Also, in our patient, diagnosis was determined after removing the mass.

In most reported cases, the patients didn’t have any problem at long follow-up after surgery (3, 9); our patient also didn’t have any problem after 2-years follow-up.
